# Processing Techniques and Applications of Silk Hydrogels in Bioengineering

**DOI:** 10.3390/jfb7030026

**Published:** 2016-09-14

**Authors:** Michael Floren, Claudio Migliaresi, Antonella Motta

**Affiliations:** 1 Department of Mechanical Engineering, University of Colorado Boulder, Boulder, CO 80309, USA; 2 Department of Industrial Engineering and Biotech Research Center, University of Trento, via Sommarive 9, Trento 38123, Italy; claudio.migliaresi@unitn.it (C.M.); antonella.motta@unitn.it (A.M.)

**Keywords:** silk fibroin, hydrogel, bioengineering

## Abstract

Hydrogels are an attractive class of tunable material platforms that, combined with their structural and functional likeness to biological environments, have a diversity of applications in bioengineering. Several polymers, natural and synthetic, can be used, the material selection being based on the required functional characteristics of the prepared hydrogels. Silk fibroin (SF) is an attractive natural polymer for its excellent processability, biocompatibility, controlled degradation, mechanical properties and tunable formats and a good candidate for the fabrication of hydrogels. Tremendous effort has been made to control the structural and functional characteristic of silk hydrogels, integrating novel biological features with advanced processing techniques, to develop the next generation of functional SF hydrogels. Here, we review the several processing methods developed to prepare advanced SF hydrogel formats, emphasizing a bottom-up approach beginning with critical structural characteristics of silk proteins and their behavior under specific gelation environments. Additionally, the preparation of SF hydrogel blends and other advanced formats will also be discussed. We conclude with a brief description of the attractive utility of SF hydrogels in relevant bioengineering applications.

## 1. Introduction

Hydrophilic polymeric networks (hydrogels) represent a suite of advanced material platforms attractive for a diversity of biomedical applications. Hydrogels are defined as a broad class of polymer networks stabilized (crosslinked) either by chemical or physical methods and dispersed throughout an immobilized water phase [1]. The hydrophilicity and stability of these polymer networks permit the penetration and absorption of water (swelling) without dissolving, thus maintaining their three-dimensional (3D) structure and function. Hydrogels are excellent material candidates for a variety of biomedical therapies, whereby water-based, soft material applications are critical. These applications include, but are not limited to, bio-responsive scaffolds, drug delivery vehicles and cell culture platforms [2].

**Figure 1:**
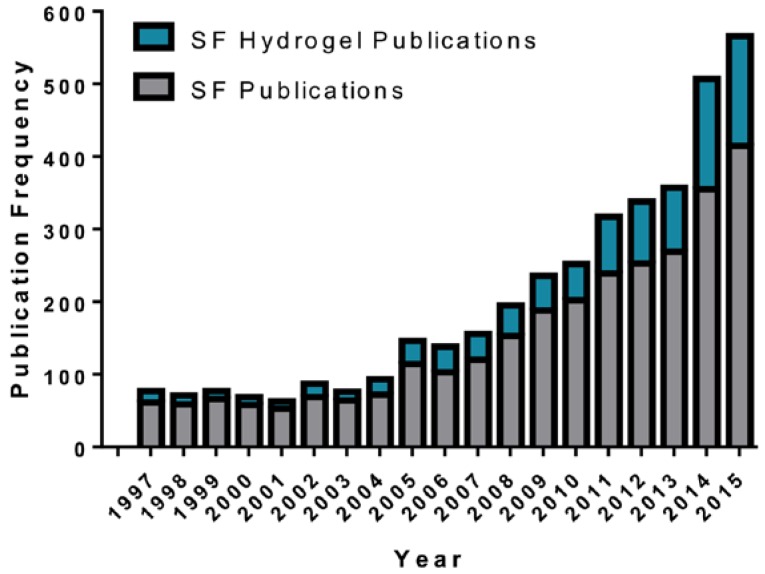
Frequency of publications related to silk fibroin (SF) (grey) and SF hydrogels (blue) by year. Data obtained by searching for SF and SF hydrogel in the Web of Science.

Material properties, such as chemistry, surface properties and biocompatibility, are significant factors that must be considered when designing hydrogels in biomedicine. Synthetic polymers are advantageous for developing hydrogel platforms as mechanical properties, and the degradation kinetics can be tailored to meet the application requirements. Specifically, synthetic polymers are attractive because they can be fabricated into various shapes with desired morphologies and features that can be permissive for cell maintenance and in-growth [3]. Synthetic polymers can be produced reproducibly with specific molecular weights, block structures, degradable moieties and crosslinking mechanism. These properties, in turn, govern the material formation dynamics, crosslinking density and material mechanical and degradation properties. However, while synthetic materials are attractive for their cost, reproducible fabrication and simple manufacturing, lack of cell-recognition sites, as well as the potential for toxic degradation products causing undesirable inflammation are often disadvantageous [4]. In contrast, in nature, fibrous proteins represent the foundation for mechanically-robust structures (i.e., collagen fibrils), provide recognition sites for cell binding and hierarchical organization, as well as provide anchoring sites for other extracellular matrix components. Among naturally-derived fibrous proteins, silk proteins have found significant utility in biomedicine due to their high biocompatibility, tunable biodegradability and material format versatility [5]. For instance, the toughness of some silks surpasses that of several performance fibers [6]. Likewise, silks from silkworms (*Bombyx mori*) are abundant in nature, and large quantities can be obtained from spun cocoons, suggesting their use as a natural polymer in several fields [7]. Perhaps its earliest biomaterial rendition, natural silk fibers have been employed as sutures for wound ligation for centuries [8]. However, the utility of silks as a biomaterial has evolved tremendously over the years to include an impressive portfolio of material formats (nets, sponges, films, gels, amongst others [5]), as well as applications (implants, tissue scaffolds, drug delivery vehicles, medical photonics [9]), which are continuously expanding (Figure 1).

The aim of this review is to illustrate the several advances and techniques used to develop hydrogels based on silk proteins. We introduce the reader to the structural and functional characteristics of this unique family of natural polymers, with emphasis on silks obtained from *Bombyx mori* silkworms. Applications and the advanced utility of silk hydrogels in several industries will also be addressed.

## 2. Characteristics of Silk Proteins

Silks are a peculiar family of biopolymers produced by several organisms as structural and multifunctional materials. Lepidoptera produce silk filaments mainly to build their cocoon (the only example in nature of a singular, long continuous filament) providing protection (from humidity, bacteria, molds, UV, etc.) during metamorphosis. Combining several types of silks with different properties, spiders make nets, used to capture food, and to protect from bacteria. Short fibers of silk are produced by bees and combined with wax during honeycomb assembly, representing a composite material. Likewise, Mollusca fabricate their “ceramic” shell starting from an organic template made by collagen and silk and further utilized in the byssus fibers (*Mytilus*). The most popular and used silk is derived from silkworm cocoons, due to its reproducibility (silkworm can eat just mulberry leafs) and availability.

Silk fibroin (SF) is a polymer with a unique hierarchical structure, multifunctional and environmentally responsive, modulating and driving its final properties. Silk proteins are characterized by a highly repetitive primary sequence, which leads to significant homogeneity in their secondary structures, imparting peculiar structural properties, in contrast to globular proteins, which are less ordered and mainly provide catalytic and molecular recognition functions [10]. Silk secondary structures permit tight packing of stacked beta sheet regions stabilized by hydrogen bonds between adjacent protein chains. These crystalline domains generate hydrophobic regions within the silk proteins and convey much of the strength and resiliency of silk materials.

Silk proteins are comprised of a main chain, heavy-chain, which can be considered a hydrophobic protein with co-block design. Hydrophilic segments are involved in the self-assembling process, resulting in changes in water content, regulating the mechanical properties of the final material. Instead, hydrophobic regions formed by the regular sequence of the hexapeptide Gly-Ala-Gly-Ala-Gly-X (were X can be Ser or Tyr or Val) are responsible for the secondary crystalline conformation that can be formed depending on processing chemical/physical stimuli [11], in particular if regenerated SF is used as the starting step, further influencing the mechanical properties. Regenerated SF conformations are one of the most important parameters that can influence the final 3D self-assembly of the protein, water correlation and mechanical properties and determining different biological outcomes [12]. In addition, two short amino acid sequences that are RGD-like are present in the N-terminal segment of heavy-chain, VTTDSDGNE and NINDFDED, and the recognized fibroblast integrin [13]. The control of the hierarchical assembling of SF is the key to design an instructive matrix with specific biological functions and physico-chemical properties for applications in the biomedical field [12]. Particularly interesting is the ability of silk fibroin-based matrices to cross-talk with stem cells, driving their specific differentiation [14,15], with the inflammatory system [16] and angiogenesis [17,18] triggering tissue repair and regeneration, as well as controlling scaffold enzymatic degradation [19]. In addition to the intrinsic properties, SF can be also chemically and functionally modified, as conjugated biological moieties, obtained with different amino acid compositions through genetic modification. New technologies are also applied to overcome spider silk limitations, first of all the availability, by recombinant production in bacteria (*Escherichia coli*), yeast (*Pichia pastoris*), insects (silkworm larvae), plants (tobacco, soybean, potato, *Arabidopsis*), mammalian cell lines (BHT/hamster) and transgenic animals (mice, goats), as plants are organisms [20].

## 3. Regulation of Silk Protein Gelation

**Figure 2:**
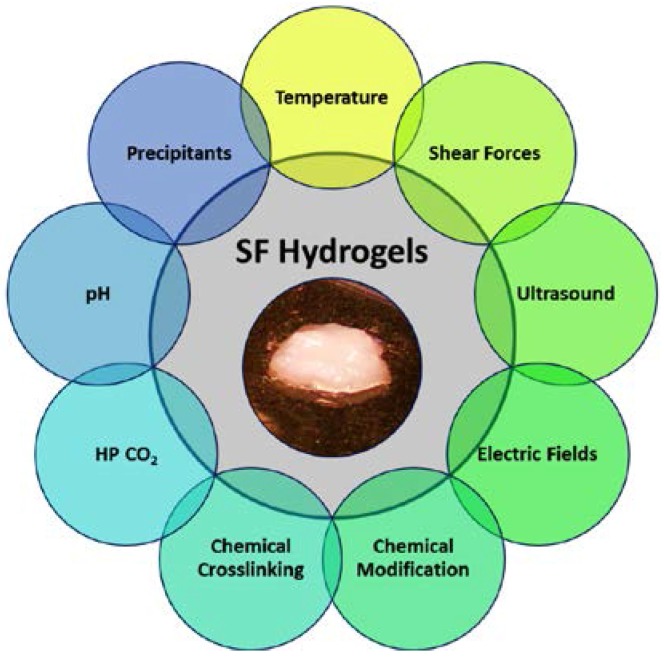
SF hydrogels can be prepared from several methods. Chemical methods: precipitants, pH, HP CO_2_, chemical crosslinking, chemical modification. Physical methods: temperature, shear force, ultrasound, electric fields.

Silk materials can be fabricated into versatile formats, specifically aqueous-based platforms, such as hydrogels, as their properties lend utility for a diversity of medical applications. The processing of silk proteins into hydrogels can be performed using a number of techniques as to be described in the following section (Figure 2). While there are several different processing methods described here, many of these techniques take inspiration from the natural silk spinning process [7]. Likewise, the preparation of natural silk materials undergoes both chemical and physical processes, similar to what is found in the native spinning process, to achieve the final stabilized fiber product. Synthetic recapitulation of these natural spinning processes, therefore, laid the foundation for the preparation of synthetic aqueous-based silk materials.

### 3.1. Chemical Methods

#### 3.1.1. Precipitating Agents

Precipitants of macromolecules classically can be divided into four broad categories: (1) salts; (2) organic solvents; (3) large polymeric agents; and (4) low molecular weight polymers [21,22].

The influence of salts in macromolecular solutions is a complex phenomenon; however, it is generally accepted that the effect of ions on protein precipitation is due to competition afforded by each constituents’ desire to bind water. This competition of the two species in solution can result either in a sum deprivation of the solvent interaction with the protein (salting out) or a sum addition of the solvent to the protein (salting in). While not strictly accurate, it is believed that ions that effectively induce protein precipitation may be thought to dehydrate the protein hydration shell, thus encouraging protein-protein association [21]. The strength and tendency of these ions to effectively dehydrate or reinforce the protein solvent layer are generally accepted to follow the Hofmeister series [23]. Accordingly, the gelation of silk proteins has been studied under various concentrated salt solutions. Kim et al. [24] studied the effect of the addition of Ca^2+^ and K^+^ ions on the gelation behavior of SF aqueous solutions. The authors reported significantly reduced gelation times for silk solutions with Ca^2+^, but minimal effects due to the presence of K^+^ ions. Interestingly, these results differed from solutions of natural spider silk dope, which under the presence of K^+^ ions displayed a rapid nanofibril formation and consequent aggregation [25]. Such differences between the two studies highlight the peculiar structural differences between the two different silk sources. In addition to hydrogels, the addition of sufficiently high ionic concentrations (salting out) allows for the preparation of silk particles. Lammel et al. [26] performed a salting out procedure using potassium phosphate to produce silk hydrogel particles with tunable properties, such as secondary structure and particle size. Unique to previous techniques, the authors adjusted the solution pH by judiciously appropriating different mixing ratios of mono- (pH 4) and di-basic (pH 9) potassium phosphate solutions, thereby allowing the fine adjustment of the solution pH between four and nine. The manipulation of the solution pH was found to have a profound effect on the silk secondary structure, zeta potential, as well as salting out efficiency, with the formation of silk hydrogels prepared in the low pH range.

Organic solvents can also be employed to influence the precipitation of proteins in solution. Alcohols are commonly utilized to induce local dehydration of α-helices structures and encouraging the transition to aggregate-prone β-sheet structures [27]. Typically, organic solvents alter the dielectric constant of the medium; a net reduction in the dielectric constant would in turn reduce the electric fields in solution, which mediate macromolecular interactions, and is an essential driving force to maintain protein solubility. Thus, the addition of an organic solvent reduces the solvating capacity of water, increasing protein-protein interactions and favoring aggregation [21]. Rammensee et al. prepared spider silk hydrogels with the addition of methanol in solution [28]. Here, the authors reported the formation of nanofibrils within the hydrogels during gelation, which contributed to the interesting mechanical properties of the aforementioned silk hydrogels. Similar to the mechanism of silk gelation demonstrated by methanol, ethanol-induced silk hydrogels have also been prepared. Numata et al., in an effort to precisely investigate the different modes of water storage within the hydrogel, namely bound water vs. free water, prepared SF hydrogels using different ratios of ethanol to silk solution [29]. The formation of β-sheet structures was confirmed by attenuated total reflection-Fourier transform infrared spectroscopy (ATR-FTIR) and wide-angle X-ray scattering (WAXD) analysis, further validating the role of alcohol as a desiccating agent, whose primary effect is to dehydrate the protein, inducing β-sheet formation and subsequent physical crosslinking of the proteins.

Surface agents also play a particular solvating role in protein-protein interactions. Surfactants can either encourage or inhibit protein aggregation depending on the nature of the surface agent, as well as the protein of study. Regarding the case of surfactant-supported protein precipitation, it is generally accepted that the binding of the surfactant to the protein results in protein unfolding and the emergence of an aggregation-prone state [30]. Accelerated silk hydrogel fabrication can be achieved using the anionic surfactant sodium dodecyl sulfate (SDS) with stable hydrogel formation observed on the order of minutes. Here, the authors attributed the rapid gelation of silk hydrogels to the strong hydrophobic forces and electrostatic effects triggered by the surfactant, leading to protein unfolding and rapid association into particles and, ultimately, an organized gel network [31].

Large polymeric precipitating agents, such as polyethylene glycols, can also influence the precipitation of proteins from solution. Unique to other molecules, polymer additives result in volume exclusion effects that can effectively encourage protein-protein interactions and subsequent aggregation. Unlike proteins, synthetic polymers do not exist in solution with a specific shape or conformation, but rather twist and entangle randomly within solution, thereby occupying more space than their protein counterparts [21]. The excessive volume occupancy observed by polymeric additives results in the deprivation of available solvent to interact with the other macromolecules (proteins) in solution, resulting in molecular partitioning and aggregation. Kim et al. prepared silk hydrogels at several concentrations of polyethylene oxide (PEO) and compared their findings with pure silk solutions dialyzed against PEG solutions [24]. Their findings revealed that silk protein gelation was facilitated by the movement of water from the silk proteins to the PEO chains, rather than through a direct interaction of the silk protein with PEO. Therefore, evidence suggests that polymeric additives have the potential to play two roles in affecting silk protein aggregation: indirect protein dehydration by volume exclusion and direct protein dehydration via the movement of water by osmosis.

The use of small neutral additives, such as polyols, can also influence the aggregation phenomenon of protein solutions. Predominantly, these small additives influence protein-protein interactions through their ionic strength or specific interactions with the individual proteins [30]. The addition of glycerol to SF aqueous solutions results in significantly reduced gelation times, as reported by Hanawa et al. [32]. Here, the authors attribute the accelerated gelation kinetics to the increased hydrophobicity of the protein from the local dehydration effects from the competition of water from the glycerol molecules. Silk hydrogels prepared using glycerol as a precipitation agent have been studied for biomedical applications [33]. The use of low molecular weight poloxamer 407 (m.w. 12,500) also sufficiently reduced the gelation time for SF solutions, which was proposed to be a result of the enhanced hydrophobic interactions between poloxamer and the silk proteins [34].

#### 3.1.2. pH

Solution pH is a parameter that can have a significant influence on protein solubility. The pH of the solution directly influences the distribution and net charge of macromolecules in solution, such as the surface charge of proteins, which ultimately governs protein-protein interactions. Theoretically, the solubility of proteins is at a minimum as the solution pH approaches the isoelectric point (pI) as a consequence of the minimal protein charge-charge repulsion. It follows that the fine adjustment of the solution pH to near to the isoelectric point, pI, of the protein of interest can be a facile tool to induce protein precipitation.

Silk hydrogels have been prepared using reduced pH protocols [24,35,36]. Ayub et al. demonstrated stable silk hydrogel formation and improved gelation kinetics by reducing the solution pH to near the isoelectric point of SF (3.8–4.0) [35]. Protonation of silk solutions leads to a net reduction of the repulsive forces between adjacent silk proteins, increasing macromolecular collisions and, thereby, encouraging physical cross-linking [36]. Mechanistically, the propensity to aggregate at reduced pH was attributed to the dominant fraction of negatively-charged (acidic) amino acid residues in the silk chain [36]. Reversible SF gelation has been observed by shifting solution pH with acidic or basic vapors for short periods of time [37]. This reversible behavior was attributed to the short exposure to acidic environments, resulting in the formation of gel networks dominated by relatively weak interactions, such as limited hydrogen bonding or hydrophobic interactions.

#### 3.1.3. Solution Acidification via Hugh Pressure CO_2_

We have demonstrated the gelation of SF aqueous solutions using high pressure carbon dioxide (CO_2_) as a volatile acid [38]. The submission of aqueous solutions to high pressure CO_2_ allows for the fine adjustment of solution pH coupled with the efficient recovery of the solvent upon venting to the atmosphere, thereby eliminating processing complexity and circumventing post treatment solvent removal protocols. To this aim, we submitted aqueous silk solutions to high pressure CO_2_ at various pressures and processing times. Using this approach, the resultant hydrogels could be prepared in relatively short periods, less than 2 h, at modest CO_2_ pressures (60 bar) [38]. The silk hydrogels prepared from this technique displayed improved porosity and physical properties when compared to a control silk hydrogel prepared from a conventional acid titration. Improved porosity and microstructure of the hydrogels are believed to be a result of a phase inversion process in which CO_2_, acting as an anti-solvent, partitions the solution into a polymer-rich and polymer-lean phase [38]. Upon removal of the CO_2_ during venting, bubble nucleation occurs, facilitating porosity and retaining the final hydrogel microstructure.

#### 3.1.4. Chemical Stabilization

**Figure 3:**
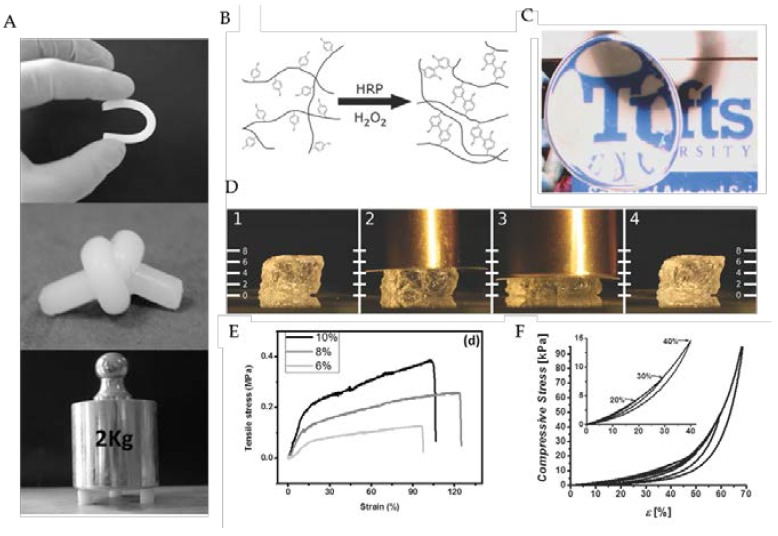
Mechanically-diverse SF hydrogels prepared by different processing techniques. (**A**) Blending SF with hydroxypropyl methyl cellulose (HPMC) produces robust mechanical prosperities highlighted visually by bending, knotting and compressing. Tensile curves prepared from SF-HPMC blend hydrogels of different concentrations (**E**). (**B**–**D**) Highly elastic hydrogels of SF have been prepared by chemically crosslinking tyrosine residues, dityrosine bonds, within SF via a horseradish peroxidase (HRP) reaction resulting in robust hydrogel networks displaying excellent elasticity and resilience. (**D**) Strain response of elastic SF hydrogel after compression with 50 g (2) and 100 g (3) brass weights and exhibiting complete recovery after removal (4). (**F**) Cyclic compression of elastic SF hydrogels reveal excellent recovery below 70% strain; the inset displays complete recovery below 40% strain. (**A**,**D**) Reproduced with permission from [64]. (**B**–**D**,**F**) Reproduced with permission from [41].

In addition to chemical additives, crosslinking agents can also be used successfully to fabricate stable SF blend hydrogels. Unlike the techniques presented earlier, which involve physical crosslinking mechanisms, chemical crosslinking agents result in the covalent linkage of protein chains and the formation of a stable hydrogel network [39]. Exploiting the significant presence of phenol groups in the tyrosine side chains of SF, approximately 5% of amino acids in SF [40], a novel method to covalently crosslink tyrosine residues in silk proteins, via horseradish peroxidase and hydrogen peroxide, has recently been explored [41]. Stabilizing SF hydrogels via covalent dityrosine bonds, rather than traditional β-sheet formation, resulted in robust hydrogel networks, lacking rigid crystalline domains and displaying excellent elasticity and resilience. The resulting hydrogels tolerated shear strains approaching 100%, fully recovered from compressive strains greater than 70%, all while exhibiting stiffness between 0.2 and 10 kPa (see Figure 3).

#### 3.1.5. Chemical Modification of SF

Methods to modify the chemical nature of SF have been explored to enhance the versatility of silk materials. While SF is quite large, containing over 5000 amino acids, chemical modification of silks is often challenging as the majority of silk proteins are composed of non-reactive amino acids, such as glycine and alanine. However, there is a significant quantity of tyrosine residues that can be modified with established chemistries [42]. For instance, a novel method to functionalize the tyrosine residues in silk was proposed using diazonium coupling chemistry [43]. By incorporating a variety of functional groups, namely sulfonic acids, carboxylic acids, ketones and alkanes, this method can impart various non-natural functional groups into SF resulting in tailorable hydrophobic and hydrophilic properties. By modifying the chemical nature of SF, β-sheet self-assembly could be inhibited (sulfonic acid groups) or augmented (hydrophobic groups), resulting in slow to rapid hydrogel formation. The authors could modulate gelation time from 5 min–2 h based on the level of tyrosine modification or SF solution concentration [43].

### 3.2. Physical Methods

Similar to the aforementioned chemical processes, several physical stimuli also play important roles in protein stability and solubility in solution. The application of physical stimuli in several formats, for instance temperature or sonication, leads to the transfer of energy within the system, ultimately affecting the hydration and folded state of the protein, both of which constitute the principal events associated with protein aggregation and assembly.

#### 3.2.1. Temperature

Arguably one of the most critical factors associated with protein aggregation [30], temperature influences the average kinetic energy of the system associated with the constituent particles (macromolecules, solvent molecules, etc.). Increasing the system temperature (kinetic energy) influences the vibration mode of molecules and thereby directly influencing species diffusion coefficients, increasing the frequency of molecular collisions and encouraging macromolecular assembly.

Regarding individual macromolecules, the physical basis for protein aggregation at increased temperatures relies on changes in the hydration of the system, which is governed by the second law of thermodynamics [44]. The enthalpy of hydrophobic hydration (ΔH_hydration_) is negative due to the increased order of water molecules encompassing the hydrophobic moiety; thus, the system favors exothermic processes. In accordance with the second law of thermodynamics, a net increase in entropy (ΔS_hydration_ becomes positive) is required to accommodate the limited Gibbs free energy of solvation (ΔG_hydration_ = ΔH_hydration_ − TΔS_hydration_) at increased temperatures. Thus, upon raising the temperature (kinetic energy) of the system, the water of hydration surrounding the hydrophobic domains of the protein becomes less ordered free water [45]. The disruption of the dense packing of water (hydration shell) encompassing the protein at increased temperatures has two primary effects on protein association: first, the local dehydration of hydrophobic moieties results in increased hydrophobic exposure, increasing hydrophobic interactions and association and, secondly, perturbing the free energy state of macromolecules, leading to protein unfolding and increased hydrophobic region exposure. The sum of these events encourages protein aggregation and assembly.

The influence of temperature on the aggregation and assembly of silk proteins has been studied previously [24,36,45]. Reports have associated significantly reduced gelation times with increased temperatures [24,36]. Matsumoto et al. reported a reduction of gelation time from 12 down to eight days for 2 wt % silk solutions at room temperature and 37 °C, respectively [36]. Increased molecular collisions [24] as well as temperature effects on hydrophobic hydration [36] represent the leading proposals to elucidate the accelerated gelation of silk proteins at elevated temperatures.

#### 3.2.2. Shear Forces

Sufficiently high shear forces acting on macromolecules can also result in significant aggregation and gelation phenomenon. The application of shear forces to polymeric solutions imposes both extensional and rotational forces to the fluid, resulting in changes to macromolecular orientation and chain stretching [46]. These changes to the molecular landscape can affect polymer chain end-to-end distances and distribution, increasing concentration fluctuations and enhancing intermolecular interactions [47]. Silk hydrogels can be prepared by applying appreciable shear forces through solution vortexing [48]. Here, it was demonstrated that the gelation kinetics could be controlled from rapid (minutes) to slow (hours) by adjusting vortex time, solution temperature and/or protein concentration. The ability to induce gelation in rapid timeframes would allow for the homogenous encapsulation of cells into the hydrogel matrix, which has been proposed for injectable cell delivery systems [49].

#### 3.2.3. Ultrasound

The application of high frequency sound waves (sonication) to macromolecular solutions can result in constituent aggregation and gelation. When a fluid is exposed to ultrasound, the sound waves create locally-expanded and compressed regions within the medium, resulting in cavitation and bubble formation [50]. Upon the subsequent collapse of these bubbles, the fluid is accelerated considerably, creating molecular collisions, which can result in local extreme temperatures and pressures [50]. The sum effect of these contributions ultimately results in local chain dehydration, encouraging macromolecular aggregation due to the increased exposure of hydrophobic domains.

Sonication of silk proteins has also been explored to control protein aggregation and hydrogel fabrication. Gelation kinetics could be controlled from minutes to hours by adjusting the sonication parameters (power output, time) as well as the initial protein concentration [51]. Silk protein gelation could be further tuned with the addition of K^+^ ions and pH adjustment, which were found to both promote gelation in concert with the sonication parameters [51]. The potential to induce gelation in minutes allowed for the encapsulation of human bone marrow-derived mesenchymal stem cells (hMSCs) into the hydrogels post-sonication. The encapsulated cells were found to proliferate up to 21 days in the hydrogels at a lower protein concentration (4 wt %), with reduced cell survival reported for the hydrogels with higher protein content [51].

#### 3.2.4. Electric Field

The use of electric fields to control silk protein gelation has recently garnered interest toward the preparation of silk hydrogels. Mechanistically, the application of electric fields presumably increases the local proton concentration at the vicinity of the positive electrode, resulting in local reduction of pH and aggregation of the silk proteins [48]. Due to the rapid gelation of silk proteins under electric fields, these novel hydrogels were afforded with several interesting features, including adhesive properties [52]. Further, it was revealed that the silk hydrogels prepared under electric currents were reversible. Upon changing the polarity of the applied voltage, stable silk hydrogels could be dissolved back into solution, whereby it would subsequently precipitate around the opposite terminal [52].

### 3.3. Blend Hydrogels

#### 3.3.1. Synthetic Polymers

Synthetic polymers are well established and commonplace in the biomedical industry in part due to their ease in modifying significant polymeric features simply by adjusting the macromolecular properties, such as molecular weight and monomer chemistry. The result of such manipulations can directly influence the physical, mechanical and degradation properties of the prepared polymer, all of which afford functionality and task-specific material traits. Consequently, synthetic polymers have gained considerable interest as biomaterials for a breadth of medical therapies, augmenting material features and serving as vital components for composite polymeric systems.

Composite hydrogels of SF and poly(vinyl alcohol) (PVA) have been fabricated by incubating aqueous mixtures of the polymers at −50 °C for 2 h, subsequent freeze-drying and rehydration [53]. The elongation at break improved significantly, both for wet and dry test specimens, with increased PVA content. Furthermore, a positive correlation between the Young’s modulus obtained from dry specimens and increased PVA content was also demonstrated. Employing repeated freeze-thaw cycles resulted in a systematic tuning of the hydrogel structure and mechanical properties.

Semi-interpenetrating polymer network (SIPN) hydrogels composed of SF and PEG have been prepared by mixing aqueous solutions of each polymer and subsequent crosslinking of the PEG macromere using ultraviolet (UV) rays [54]. The thermal behavior measured by differential scanning calorimetry (DSC) demonstrated a decreased degradation temperature for the silk component of the composite hydrogel, suggesting the formation of a miscible blend and SIPN formation. Due to the hydrophilic properties of the PEG macromere, the water content of the silk hydrogels increased considerably with the addition of PEG content. Further, it was revealed that the tensile strength and elongation at break increased significantly for the SIPNs when compared to the neat silk and non-crosslinked silk/PEG hydrogels and increased with PEG content. Later, SIPN hydrogels replacing PEG with poloxamer 407, having an acrylate-terminated PEO derivative, and silk were proposed in order to enhance the mechanical and functional properties of SF. Utilizing a similar technique as before [54], the silk/poloxamer SIPN hydrogels were prepared by simply mixing the two aqueous polymer solutions and subsequently crosslinking the poloxamer macromere under UV light [55]. The improved biological response associated with poloxamer 407 for wound healing therapies was believed to offer a significant improvement over the silk/PEG SIPN systems.

#### 3.3.2. Natural Polymers

Natural polymer demand is expected to grow in several industrial fields, including the biomedical one; as a result, several composite hydrogels systems combing silk proteins with other natural polymers have become popular. Controllable in vivo degradation rates and improved mechanical features of silk materials suggest silk proteins as an attractive constituent for composite natural polymer hydrogels. Likewise, versatile gelation schemes, as detailed in previous Section 3.1 and Section 3.2, allow for a diversity of facile methods to prepare natural polymer composites comprised of silk proteins.

Hydrogel composites of gelatin and SF have been proposed to improve thermal stability and mechanical properties to around body temperature (37 °C), whereby gelatin alone leads to dissolution [56]. The inclusion of beta-sheet-rich silk domains served to stabilize the hydrogel network and extend the solid-like characteristics of the hydrogels to temperatures above the established helix-coil transition of pure gelatin (30 °C) [56]. Chemical methods have also been employed to prepare SF-gelatin blend gels. Sun et al. proposed a method to stabilized SF-gelatin blend gels by crosslinking with the natural compound genipin [14]. Along these lines, Hu et al. combined methacrylated gelatin (GelMA) with SF to prepare photocrosslinkable blend hydrogels [57]. The use of a methacrylate photopolymerization step allowed for the rapid stabilization of these SF-GelMA blend hydrogels under UV, which were suggested for photolithography or cellular encapsulation [57].

Pure collagen hydrogels are generally soft, with an elastic moduli ~2.7 kPa [58], as well as degrading more rapidly in vivo through recognized enzymatic pathways compared to synthetic hydrogel counterparts [59]. SF-collagen blend hydrogels have successfully been developed using a chemical crosslinking method via 1-ethyl-3-(3-dimethylaminopropyl) carbodiimide hydrochloride (EDC) [60]. The prepared SF-collagen hydrogels demonstrated improved mechanical and thermal properties when compared to pure collagen matrices.

Hydrogel blends of SF and hyaluronic acid (HA) have also been prepared by physical stabilization via ultrasonication [61]. A critical component of the extracellular matrix (ECM), HA is widely distributed throughout various tissues and is believed to contribute significantly to cell proliferation and migration [62]. However, pure HA hydrogels typically require chemical methods to stabilize [63], as well as display limited degradation tuning. Blending SF with HA improved the mechanical properties of the prepared hydrogels, providing a greater suite of applications that demand better material handling and degradation/release kinetics [61].

Blending of SF with cellulose ethers has also been explored. Hydroxypropyl methyl cellulose (HPMC), a cellulose derivative, displays a lower critical solution temperature (LCST) at approximately 62 °C, whereby heating above the LCST exposes hydrophobic groups. Mixing and heating of SF-HPMC blends results in robust hydrogel formation catalyzed by the formation of β-sheets induced by hydrophobic interactions between HPMC molecules and SF upon heating [64]. Employing this method, SF-HPMC blends with impressive mechanical properties where produced, with a tensile modulus reported greater than 1.0 MPa, as well as a break energy approaching 3500 J/m^2^, comparable to several natural tissues, such as skin and cartilage (Figure 3). The authors proposed a gelation mechanism whereby the hydrophobic interactions of SF and HPMC resulted in uniformly-dispersed physical crosslinks associated with smaller β-sheet structures, ultimately contributing to the superior mechanical properties of the blend hydrogels [64]. Gong et al. further exploited the unique hydrophobic interactions of SF and hydroxypropyl cellulose (HPC) to produce injectable hydrogels with thixotropic properties [65]. By elevating the SF-HPC solution blend above the LCST, phase separation occurred resulting in SF-rich domains, β-sheet formation and subsequent gelation. As the phase separation proceeds, the phase-separated microstructure coarsens, ultimately leading to a percolated hydrogel network. The unique structure and interaction of the SF-HPC blend gels exhibited thixotropic properties with cell encapsulation potential [65].

Alginate is a natural polysaccharide derived from algae that has wide applications in the biomedical industry. In the presence of calcium chloride (CaCl_2_), sodium alginate rapidly gels, allowing for encapsulation of cells with minimal negative impact [66]. However, alginate hydrogels dissolve without the presence of divalent ions; therefore, their use for extended in vivo applications is limited. Ziv et al. sought to exploit the rapid gelation properties of alginate with the improved degradation properties of SF for improved stem cell maintenance and transplantation [67]. By blending the two natural polymers, the authors improved the gelation kinetics with tailorable mechanical and structural properties while maintaining biocompatibility. Furthermore, incubation of the prepared SF-alginate blend hydrogels with fetal bovine serum (FBS) containing CaCl_2_ allowed for temporal mechanical tuning of the hydrogel substrates during cell culture, which was proposed to help regulate stem cell differentiation [67].

Chemical crosslinking is often a useful alternative when the manipulation of protein solubility is complex or undesirable, as in the case of solvent-intensive gelation protocols. These scenarios occur commonly during the preparation of hydrogel blends where difficulty arises from uncommon crosslinking mechanisms amongst the two polymer constituents; the solution to which can often be realized through the employment of chemical crosslinking agents. SF-chitosan blends have successfully been chemically crosslinked with genipin, a natural crosslinking agent extracted from gardenia fruit [68], which targets the primary amine residues within the SF and chitosan, yielding composite hydrogels [69]. The addition of SF improved the mechanical response of the prepared hydrogels; however, hydrogels with greater chitosan fraction demonstrated greater cell viability, highlighting the salient features of each natural polymer. Genipin-crosslinked SF-gelatin blend hydrogels have also been proposed recently [14]. By adjusting the ratio between gelatin and SF, the morphology, structure and mechanical properties of the hydrogel scaffolds could be modulated. The SF-gelatin blend hydrogels promoted cell proliferation for short- and long-term cultures, as well as supported the epithelial ectodermal differentiation fate of pluripotent stem cells [14].

### 3.4. Genetically Engineered Silks for Hydrogel Formation

In nature, biomolecules, such as peptides and proteins, organize into complex structures that are accredited to functionality [70]. Inspired by this elegant natural phenomenon, researchers have begun to exploit the self-assembly of these components towards engineering synthetic, novel supramolecular architectures. For instance, through genetic manipulation, protein templates may be engineered at the molecular level, allowing for robust self-assembly processes [71]. Silk-elastin-like protein polymers (SELPs) represent a family of genetically-engineered biomaterials with interesting self-assembly properties. By combining silk-like (Gly-Ala-Gly-Ala-Gly-Ser) with elastin-like (Gly-Val-Gly-Val-Pro) peptide sequences, along with the appropriate composition, SELPs undergo an irreversible gelation, which can be accelerated via other physical stimuli, such as temperature [72,73]. The stabilization of SELPs is believed to occur through extensive hydrogen bonding amongst the silk-like blocks, resulting in robust crosslinks throughout the network. However, the inclusion of the more ‘elastic’ elastin-like blocks imparts flexibility and improved water solubility of the polymer network [74]. The culmination of these properties allows for the production of robust silk-like materials with the elastic properties often found in elastic protein assemblies. Undoubtedly, these genetically-engineered silks will provide the novel structural and functional complexity required for many future biomaterial platforms [75]. Nonetheless, a discussion of these advanced silk materials is beyond the scope of this review; we refer the reader to several excellent reviews on genetically-engineered silks and their unique material features [75,76].

## 4. Silk Hydrogels in Bioengineering and Medicine

### 4.1. Tissue Engineering and Regenerative Medicine

**Figure 4:**
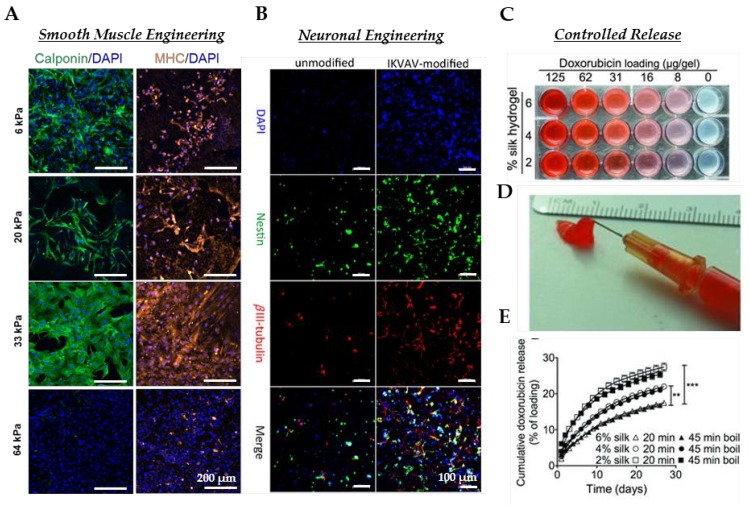
Applications of SF hydrogels in bioengineering. (**A**) hMSCs cultured on SF hydrogels of different stiffness with 10 ng/mL TGF-β1 for 72 h for selected vascular SMC markers: calponin (green), myosin heavy chain (MYH11) (orange). Scale bar: 200 μm. (**B**) hNSCs encapsulated in unmodified and IKVAV-modified SF hydrogels after seven days. Cells were stained with Nestin (green), βIII-tubulin (red); bars = 100 μm. (**C**) Image of self-assembling SF hydrogels loaded with different amounts of the anticancer drug doxorubicin (red). (**D**) SF-doxorubicin hydrogel loaded syringe displaying injectability for clinical use. € Influence of SF hydrogel processing and cocoon degumming parameters on the cumulative doxorubicin release into PBS. Statistical analysis was performed by comparison with SF 6 wt % hydrogel; ** *p* < 0.001, *** *p* < 0.0001. (**A**) Reproduced with permission from [15]. (**B**) Reproduced with permission from [82]. (**C**–**E**) Reproduced with permission from [83].

Tissue engineering and regenerative medicine seek to provide therapeutic solutions to repair or replace damaged tissue [77]. Traditionally, tissue engineering has relied on cultivating a cell source, usually a biopsy of the host tissue, onto a structural matrix (scaffold) and subsequently implanted at the site of defect. The ability to design scaffolds that imitate the natural cellular micro-environment of the replacement tissue is vital for the success of such implants and usually involves the fine-tuning of engineered matrices on various scales. This section is dedicated to the review of SF hydrogels for tissue engineering applications, tabulated in Table 1 and illustrated in Figure 4.

#### 4.1.1. Hydrogels for Hard Tissue Engineering

##### 4.1.1.1. Bone

Bone is a mineralized tissue with hierarchical organization on several scales, imparting many of the unique physical and mechanical properties bone tissues exhibit [84]. However, similar to other tissues in the body, bone tissue cannot self-heal in the context of large defects or complete loss of tissue and must be met with surgical intervention. To resolve this, bone scaffolds, also colloquially referred to as bone ‘fillers’, have emerged to assist in the development and regeneration of new bone tissue [85]. Hydrogels based on SF have been investigated for injectable bone filling scaffolds. Fini et al. explored the bone remodeling capability of injectable SF hydrogels, prepared from citric acid titration, into critical-sized defects of rabbit femurs compared against poly(lactide-co-glycolic acid) (PLGA), a commercially available and FDA-approved polymer [78,86]. Here, the authors measured bone defect healing rate and quality of the newly-formed bone in vivo by measuring several metrics associated with bone tissue, such as trabecular bone volume, thickness, number, separation, mineral apposition rate (MAR) and bone formation rate. Bone regeneration observed in the SF hydrogel-treated defects displayed significantly higher trabecular bone volume, thickness, MAR and bone formation rate as compared to the PLGA control. Further evaluation of the regenerated bone of the SF hydrogel-treated defects after 12 weeks revealed bone properties more aligned to normal bone tissue than that of the PLGA-treated defects. The authors concluded that bone defects treated with injectable SF hydrogels resulted in accelerated bone regeneration when compared to the synthetic (PLGA) control.

Combining with growth factors, SF hydrogels have also been shown to be excellent candidates for bone regeneration. Often, growth factors are needed to improve the cellular recruitment, penetration and activity of scaffolds upon implantation in vivo. To accelerate bone remodeling in defects in the rabbit maxillary sinus, Zhang et al. loaded vascular endothelial growth factor (VEGF_165_) and bone morphogenic protein 2 (BMP-2) into SF hydrogels prepared by sonication [49]. Believed to play a critical role in new bone formation, VEGF165 and BMP-2 are key regulators involved in angiogenesis and osteogenesis, respectively [87]. The benign processing method, sonication, preserved the functionality of the loaded growth factors, which could easily be lost upon using more invasive gelation schemes, such as temperature or pH adjustment. Bone remodeling was evaluated at four and 12 weeks after in vivo implantation. At four weeks, SF hydrogel with VEGF165 only demonstrated enhanced tissue infiltration, whereas SF hydrogel with both VEGF165 and BMP-2 exhibited slightly greater bone area compared to other groups. However, at 12 weeks, the SF hydrogels combined with both factors (VEGF165 and BMP-2) displayed the largest new bone formation when compared to SF hydrogel groups containing a single growth factor or neither. The authors concluded that injectable SF hydrogels are minimally invasive, permissive to deliver multiple growth factors and can successfully regenerate bone tissue in irregular bony cavities.

##### 4.1.1.2. Cartilage

Cartilaginous tissue is ubiquitous throughout the body and particularly rich within the joints where they primarily function to dampen mechanical loads [88]. Cartilage is avascular and, as a result, the natural healing, even of modest injuries, is likely met with insufficient outcomes, and severe injuries usually require surgical resolution [89]. The high incidence of cartilage-related injuries, as well as the lack of self-healing properties of cartilage has led to a profound interest in the tissue engineering community to provide cartilage repair or regeneration therapies. Cartilage regeneration has been evaluated using SF-based hydrogels. Utilizing a sonication gelation method, Chao et al. encapsulated primary calf chondrocytes into various formulations SF hydrogels, by changing silk protein concentration, and compared against cell culture in porous silk scaffolds [79]. Here, the authors reported the proliferation and chondrogenic phenotype of primary chondrocytes encapsulated with the SF hydrogels. By adjusting the silk protein concentration of the hydrogels, the authors were capable of preparing hydrogels that approached sufficient mechanical properties for cartilage. Interestingly, the authors determined that the properties of their SF hydrogels compare well with that of agarose, which is commonly accepted as a gold standard material for cartilage tissue engineering [90].

Attempting to improve the mechanical nature of SF hydrogels for cartilage regeneration, Yodmuang et al. combined silk fibers with SF hydrogels (SF-fiber hydrogel) with the aim to mimic the intrinsic fiber morphology found in native cartilage [80]. The authors reported an equilibrium modulus in the native tissue range of cartilage for SF-fiber hydrogels, which closely mimicked collagen fiber and the proteoglycan composite architecture of native cartilage. Encapsulation of the SF-fiber hydrogels with primary bovine chondrocytes was evaluated up to 42 days and resulted in enhanced cartilage matrix deposition. The SF-fiber hydrogel constructs were suggested for more extensive in vivo testing.

Blend SF hydrogels have also emerged as potential candidates for nucleus pulposus (NP) regeneration. Preparing SF and HA blend hydrogels via sonication has been shown to support encapsulation of human chondrocytes [81]. The addition of silk improved the mechanical properties, while HA preserved sufficient swelling of the gels. Further, the authors demonstrated that the addition of SF to HA gels reduced the degradation of the gels while maintaining NP-like chondrogenic cell growth.

#### 4.1.2. Hydrogels for Soft Tissue Engineering

##### 4.1.2.1. Neuronal Regeneration

Apart from other organ or tissue systems, the nervous system has a limited ability to repair or regenerate from traumatic injury [91]. Damage to the central nervous system (CNS), for instance, is particularly burdensome by disrupting functional motor and/or sensory pathways, which contribute to a diversity of physiological systems within the body [92]. As a result, neuronal tissue engineering has relied on combining potent regenerative cells, such as neuronal stem cells (NSCs), with a sufficient scaffold for transplantation to support neural cell viability and differentiation [93].

Injectable, SF hydrogels with encapsulated NSCs have been developed with the aim to support brain regeneration [82]. SF hydrogel substrates were produced by sonication and physically and chemically (by using genipin as a cross-linker) blending with gelatin at different ratios. Mouse embryonic stem cells (mESCs) were seeded on the hydrogel substrates and kept in knockout serum replacement (KSR) for 15 days. Immunocytochemistry and QRT-PCR were performed to characterize the neural differentiation of the mESCs at Day 15. By culturing ESCs on these hydrogel substrates in KSR supplemented medium for 15 days to induce neural differentiation, it was assessed that the blended gelatin/SFs GS95 and GS80 were able to change ESCs differentiation fate from neural ectodermal to epithelial ectodermal fate compared to tissue culture plastic (TCP) [14]. Later, to improve the neuron differentiation, SF was chemically modified with the peptide laminin-derived IKVAV. To evaluate the unmodified and IKVAV-modified hydrogels, human neural stem cell were encapsulated in both hydrogels; the viability and neural differentiation were then evaluated [82].

##### 4.1.2.2. Vascular Regeneration

Cardiovascular disease (CVD) is the leading cause of death in the United States associated with one out of every four deaths, as reported in 2009 [94]. Current therapies rely on surgical intervention using autologous vascular bypass grafts; however, these therapies are often limited by surgical harvest or the disease state of the target explant [95]. Stem cell therapy promises to revolutionize the treatment of vascular diseases with the potential to regenerate vascular tissues in vitro or in vivo [96]. However, despite significant progress in regenerating vascular tissues using MSCs and other stem cells, there are still considerable challenges associated with the production of vascular phenotypes with high cell specificity, maturity and functional behavior.

Our group previously developed a technique to produce porous, SF hydrogels with tunable stiffness and morphology using the green solvent, carbon dioxide (CO_2_) [38]. Hydrogel elastic moduli approaching soft tissues (E = 6–30 kPa), combined with the ease of fabrication and biocompatibility, motivated us to use these SF materials as a platform to instruct stem cell differentiation towards the vascular smooth muscle cell (SMC) lineage in a precise manner. To achieve this, we combined the salient features of SF hydrogel (substrate stiffness) with transforming growth factor β1 with the aim of correlating these effects on the vascular commitment of human mesenchymal stem cells (hMSCs) [15]. The transforming growth factor β (TGF-β) family is a potent regulator of several cell functions, such as proliferation and spreading [97], and is strongly associated with vascular smooth muscle cell (vSMC) differentiation of stem cells [98]. One advantage to employing SF as the hydrogel material of choice was the inherent biocompatibility and biorecognition exhibited by silks. When cultured with serum-starved media upon SF hydrogels, hMSCs attached and spread for up to 72 h. The ability to culture stem cells on SF hydrogels without serum proteins allowed for the independent study of different physical and chemical stimuli. Upon combining TGF-β1 with SF hydrogels of different stiffness, we revealed that hMSC differentiation into mature smooth muscle cells can be achieved within modest culture periods (72 h) [15].

### 4.2. Controlled Release

Silk hydrogels have been suggested for several drug release schemes, including vitamin derivatives [32], buprenorphine [99], chemotherapeutic agents [83] and model biomolecules [100]. Blend hydrogels of glycerol and SF have been studied for the release behavior of the vitamin B1 derivative benfotiamine (BTMP) [32]. Adjusting the ratio of SF to glycerol, the authors revealed tunable BTMP release kinetics, which could be retarded by increasing the SF concentration, stiffness or glycerol content. Hydrogels prepared from SF of different molecular weights also have significant effects on biomolecule release kinetics. Fang et al. studied the release of buprenorphine, nalbuphine, fluorescein isothiocyanate (FITC) and FITC-labeled dextran (FD) from SF hydrogels prepared either with low m.w. protein (~18 kDa) or high m.w. protein (76 kDa). The authors found that SF hydrogel material properties and drug release kinetics could be regulated by adjusting silk protein concentration or by blending at different ratios of low and high SF protein molecular masses [99]. Chemotherapeutic agents, such as doxorubicin, have been successfully loaded into self-assembling SF hydrogels, illustrated in Figure 4. By adjusting different properties of the SF hydrogels, control over doxorubicin release could be tuned to minimize harmful side effects, yet maximize the therapeutic impact [83]. In vivo studies of SF-doxorubicin hydrogels injected locally displayed excellent antitumor response in mice and outperformed when the equivalent amount of doxorubicin was delivered intravenously [83]. Comparing the controlled release of several compounds, SIPNs of SF and polyacrylamide have also been proposed as effective drug release hydrogels. Sustainable release of two model compounds of different sizes, trypan-blue and FITC-inulin, was demonstrated, highlighting the unique features of SIPNs to control release kinetics [100].

## 5. Future Directions and Recommendations

Hydrogels prepared from SF have shown increasing utility in bioengineering over the years due to their excellent biocompatibility, mechanical tuning and versatile format preparation. With the advancement of novel processing techniques and multi-component integration, SF hydrogels are an exciting and promising lead for future biomaterial platforms. This review has provided key insights into the advanced techniques to prepare SF hydrogels with novel structural and functional features with expanding applications in tissue engineering and drug release. Fine-tuning SF gelation techniques to better control hydrogel functional features, as well as integration with single or multi-components, will certainly drive the future of SF hydrogel innovation. However, while there has been tremendous advancement in SF hydrogel technology, several limitations still require attention before these materials can reach their full potential.

Recently, hydrogels with both spatial and temporal control of physical or biochemical stimuli have been proposed as ideal ‘smart’ biological platforms. Indeed, growing evidence suggests that biological environments undergo a plethora of signaling events and that these processes are not static, but rather dynamic in time. Indeed, recent studies have suggested a significant regulatory role of stem cell fate commitment based off of the temporal expression of matrix physical cues, such as rigidity [101]. The concept that past physical events may be ‘stored’ within the cell as a repository of information later to guide the cells’ fate has actively led towards the fourth dimension (temporal control) of biomaterial design. Thus, advancements in the temporal control of SF hydrogel functional features will undoubtedly be of significant novelty in the coming years. The ability to fine-tune biochemical release or hydrogel mechanical features temporally would provide an impressive suite of applications for SF hydrogels.

In addition to improved temporal and spatial control of SF hydrogel features, the incorporation of selective biological moieties and/or ligands is also of particular interest. While SF is a well-tolerated, biocompatible biomaterial, lack of specific bioactive ligands, such as RGD, limit its potential for specific biological recognition. Biological ligands can improve cellular adhesion [102], cellular processes [103], as well as permit or abrogate disease progression [104]. The design of hydrogel platforms that instruct specific cellular events through highly tailored ligand signaling will likely be critical for future biomaterial design. The ability to incorporate these bioactive sites into SF has yet to be sufficiently studied. Improved knowledge on the interaction of SF molecular structure with specific chemical modification schemes would greatly advance our understanding and success to modify SF with biological moieties.

Lastly, the role of SF hydrogels in advanced medical diagnostics and devices has yet to see its full potential. Zhao et al. recently demonstrated SF hydrogel-based microfluidic systems using a simple multilayered fabrication process [105]. The benign fabrication process, aqueous-based at ambient temperature, permits the incorporation of biological compounds, such as enzymes or living cells, which would be labile to other fabrication processes. The ability to monitor such biological events, either as a diagnostic device or via lab on a chip platforms, can offer tremendous opportunities for future biomedical devices. Advanced hydrogel post-processing techniques could also foreseeably expand the utility of SF in biomedical devices. Recently, micrometer-scale structures have been created within bulk SF hydrogels using multiscale laser machining [106]. Beginning with preformed SF hydrogels, internal structures, voids, could be created via multiphoton absorption. Here, the authors reported approximately ten-fold improvement in laser penetration depth due to the optical clarity of SF. The post-tuning of SF hydrogel internal structures was suggested as an ideal scaffold candidate for advanced tissue engineering or biomedical device designs. While there have been leaps forward in the translation of SF hydrogels into diagnostics and biomedical device design, much work is needed to further highlight the salient features of SF biomaterials in this industry.

## Figures and Tables

**Table 1:** SF hydrogels have been developed for several tissue engineering applications.

SF Hydrogel Fabrication	Target Regeneration	Methods	Comments	Ref.
SF-gelatin blend prepared by sonication and chemical crosslinking by genipin	Neuronal tissue	Mouse embryonic stem cells (mESCs) were seeded and kept in knockout serum replacement (KSR) for 15 days	Blended gelatin/SFs were able to differentiate ESCs from neural ectodermal to epithelial ectodermal fate compared to tissue culture plastic (TCP)	[14]
SF hydrogel prepared by CO_2_ treatment	Vascular Smooth muscle	Culturing of hMSCs within SF hydrogels of variable stiffness and combined with 10 ng/mL TGF-β1	Upregulation of mature vascular smooth muscle cell phenotype (myosin heavy chain expression) of hMSCs with appropriate SF gel stiffness and growth factor within 72 h	[15]
VEGF_165_ and BMP-2 growth factors were encapsulated in 1 mL of 5.0 wt % SF solution and stabilized via sonication (25% amplitude) for 30 s	Bone	Evaluate in situ forming SF hydrogels combined with dual growth factors for rabbit maxillary sinus floor augmentation	VEGF_165_ and BMP-2 release from injectable SF gels promoted angiogenesis and new bone formation Combined use of VEGF165 and BMP-2 augmented bone regeneration	[49]
Addition of 1 M citric acid to 2% *w*/*v* SF water solution and treated at 50 °C overnight	Bone	In vitro and in vivo response of injectable SF hydrogels toward osteoblast culture and implantation in critical-size defects of rabbit distal femurs	Significant increase in TGF-β1 secretion was found for SF hydrogel compared to control SF hydrogel accelerated bone remodeling processes in vivo compared to control	[78]
Sonicated SF hydrogels of various protein concentration	Cartilage	Encapsulated primary calf chondrocytes into SF hydrogels of different protein concentration and compared against a porous silk scaffold control	Proliferation and chondrogenic phenotype maintained by primary chondrocytes encapsulated within SF hydrogels Adjusting silk protein concentration prepared hydrogels mechanical properties similar to cartilage	[79]
Composite hydrogel combining silk microfibers with sonicated SF hydrogel	Cartilage	Prepared SF-fiber blend hydrogels to mimic fiber morphology found in native cartilage	Equilibrium modulus in the range of cartilage Encapsulation of primary bovine chondrocytes up to 42 days resulted in enhanced cartilage matrix deposition	[80]
SF-HA blend hydrogel prepared by sonication	Nucleus Pulpous (NP)	Encapsulation of human chondrocytes	Addition of SF improved mechanical properties, while HA preserved swelling Improved degradation of the gels was observe while maintaining NP-like chondrogenic cell growth	[81]
SF modified with IKVAV peptide and stabilized by sonication	Neuronal tissue	Encapsulation of human neural stem cells	SF hydrogel modified by IKVAV peptide displayed improved cell viability and enhanced neuronal differentiation capability	[82]
